# Overwintering Honey Bee Colonies: Effect of Worker Age and Climate on the Hindgut Microbiota

**DOI:** 10.3390/insects12030224

**Published:** 2021-03-05

**Authors:** Patrick W. Maes, Amy S. Floyd, Brendon M. Mott, Kirk E. Anderson

**Affiliations:** 1 Graduate Interdisciplinary Program in Entomology and Insect Science, University of Arizona, P.O. Box 210036, Tucson, AZ 85721, USA; pmaes@email.arizona.edu; 2 USDA-ARS Carl Hayden Bee Research Center, 2000 East Allen Road, Tucson, AZ 85719, USA; amy.floyd@ars.usda.gov (A.S.F.); brendon.mott@ars.usda.gov (B.M.M.)

**Keywords:** honey bees, dearth, microbiota, bacteria, climate, diutinus phenotype aging, gut, health, longevity

## Abstract

**Simple Summary:**

The honey bee is managed worldwide for use in pollinating crops and producing honey. Healthy overwintered colonies are paramount to meeting spring agricultural pollination demands, but beekeepers typically report high rates of winter colony loss. Although many factors affect winter survival, the gut microbiome, demonstrated to facilitate healthy metabolism and physiology, is understudied in this context. Here, we investigate how overwintering climate (warm versus cold) alters the honey bee gut microbiome. In both climates, the gut bacteria were generally stable during overwinter. However, microbiota changes in the warm climate suggest compromised host physiology. The abundance of fungus increased two-fold in the warm climate and was strongly associated with potentially harmful bacteria. The life expectancy of worker bees in warm climates was low compared to that known for cold climates. Our results indicate that colony loss in warm climates is associated with shortened life expectancy of overwintering workers, and alterations in the gut microbiome. We suggest that overwintering in warm climates can worsen preexisting conditions of disease, parasites, and poor nutrition, increasing winter colony loss. Ultimately, our results provide new insights into overwintering honey bee management strategies.

**Abstract:**

Honey bee overwintering health is essential to meet the demands of spring pollination. Managed honey bee colonies are overwintered in a variety of climates, and increasing rates of winter colony loss have prompted investigations into overwintering management, including indoor climate controlled overwintering. Central to colony health, the worker hindgut gut microbiota has been largely ignored in this context. We sequenced the hindgut microbiota of overwintering workers from both a warm southern climate and controlled indoor cold climate. Congruently, we sampled a cohort of known chronological age to estimate worker longevity in southern climates, and assess age-associated changes in the core hindgut microbiota. We found that worker longevity over winter in southern climates was much lower than that recorded for northern climates. Workers showed decreased bacterial and fungal load with age, but the relative structure of the core hindgut microbiome remained stable. Compared to cold indoor wintering, collective microbiota changes in the southern outdoor climate suggest compromised host physiology. Fungal abundance increased by two orders of magnitude in southern climate hindguts and was positively correlated with non-core, likely opportunistic bacteria. Our results contribute to understanding overwintering honey bee biology and microbial ecology and provide insight into overwintering strategies.

## 1. Introduction

Overwintering colony health and survival is a major concern for the beekeeping industry [1]. Honey bee colonies adapted to temperate climates undergo four general stages during their yearly life cycle. In the spring, worker numbers expand exponentially as colonies forage and consume abundant resources. Colonies reaching critical mass enter a colony-level reproductive phase wherein new colonies establish through swarming. Colonies then hoard resources necessary for winter survival, including extensive honey stores to fuel colony thermoregulation [2]. Depletion of pollen stores essential for brood rearing initiates a physiological transition of young workers into a distinct long-lived (diutinus) phenotype [3]. The diutinus phenotype acts as a storage vessel for fats and proteins destined to nourish the first spring cohort. This colony-level overwintering strategy depends largely on the production and conservation of the yolk precursor vitellogenin (Vg), a glycol-lipoprotein highly expressed in adult diutinus workers. Diutinus bees accumulate Vg, which extends their life-span and improves their tolerance to starvation, disease, and oxidative stress [3,4,5]. The evolution of this life history strategy has allowed honey bee colonies to endure seasonal changes in forage availability and inclement weather by storing simple sugars in the hive as an external energy source and fats and proteins, in the form of vitellogenin (Vg), within the bodies of diutinus workers [6,7,8,9]. While workers during the growth phase live an average of 21 days, diutinus workers can live 150+ days in cold climates [10].

Honey bee colonies are managed throughout the world and subsequently spend the winter months in highly variable climates [11]. Temperature is a primary factor affecting larval development of honey bees. Prolonged exposure to cold temperature leads to higher larval mortality, with a reduced life expectancy for those surviving to adulthood [12]. To mitigate these deleterious effects, the life history strategy of temperate evolved honey bees involves the cessation of brood rearing in cold climates. This not only preserves limited nutrient stores but also provides a break in the larval disease cycle and suppresses the *Varroa* mite, the major parasite of developing brood. While immunity and antioxidant expression increases from early to late winter [13], differences in immunity profiles between summer and winter bees suggests evolutionary adaptations to both conserve energy and resist bacterial pathogens overwinter [8,14]. Consistent with these evolutionary adaptations, beekeepers have found success overwintering honey bee colonies indoors under controlled cold climate conditions of 7 °C and 25% relative humidity [15,16]. Thermoregulation by the colony is constant under these conditions, and the bees do not leave the colony for many months. In sharp contrast, thermoregulatory clustering is discontinuous in southern climates, and temperature fluctuations throughout the day allow for extensive foraging activity and brood rearing at low levels. Many southern ecosystems still provide a small amount of pollen and nectar in the winter environment, such that pollen collection and brood production do not stop. Developing broods are the preferred niche of the *Varroa* parasite and, as such, brood production in southern climates supports the proliferation of the parasitic mite, a major vector of a debilitating deformed wing virus (DWV) [17,18,19]. Ultimately, southern overwintering requires more extensive colony management that includes supplemental nutrition and timely treatment for *Varroa* mite infestations.

Mounting evidence indicates that the honey bee hindgut microbiome has a major effect on central host metabolism, increasing health and longevity [20,21,22,23,24,25,26,27]. Indeed, honey bees that lack a hindgut microbiota and consume pollen have significantly reduced Vg expression, suggesting that the microbiome is critical to central host metabolism [23]. The honey bee harbors a simple and taxonomically predictable hindgut microbiota. Regardless of sampling space and time the hindgut microbiome of adult worker honey bees is consistently dominated by six bacterial phylotypes. Core hind gut bacteria *Lactobacillus* Firm5, *Lactobacillus* Firm4 and *Bifidobacterium asteroides* are associated with the rectum while *Lactobacillus* Firm5, *Snodgrassella alvi*, *Gilliamella apicola* and *Frischella perrara* are associated with the ileum/pylorus [28,29]. Found less frequently and in lower abundance in the worker hindgut are *Bartonella apis*, *Bombella apis* [30,31] and *Lactobacillus kunkeei*, the latter two being more commonly associated with the hive environment, mouthparts, larvae and queens [20,21,22,23,26,27,28,29,30,31,32,33,34,35]. Although the gut microbiota of workers, queens and larvae have been studied, the gut microbiota of the diutinus phenotype has been largely overlooked. Recently however, Kešnerová et al., 2019 report dominance of *Bartonella apis* in the overwintering diutinus hindgut microbiota [36].

To further our understanding of how climate affects the microbial succession of diutinus workers overwinter, we test two hypotheses: (1) honey bees experiencing forage dearth and cooler temperatures in southern climates will express the diutinus phenotype, and (2) the overwintering climate will affect the hindgut microbiome. We test the first hypotheses in a southern climate under forage dearth conditions using a known age cohort to determine worker longevity and associated hindgut microbiota size and structure as workers age during winter. We test the second hypothesis by comparing hindgut microbiota changes from early to late winter associated with both a warm southern climate and cold indoor climate controlled environment.

## 2. Materials and Methods

### 2.1. Known Age Cohort

We explored hindgut microbial succession and worker longevity overwinter using a marked bee cohort. We marked approximately 2000 newly emerged worker bees with a dot of paint on their thorax, then introduced them to four different observation hives made of Plexiglas. The hives were fitted with small access doors to provide non-intrusive sampling of known age bees. These marked individuals of “known age” (KA) were initially sourced from the frames of 20 healthy *Apis mellifera* subsp. *ligustica* colonies containing late-stage pupae. Frames containing late-stage pupae were first placed in a humidity (50%) and temperature-controlled (35 °C) room, where adult bees emerged naturally from natal frames over a period of ≤24 h. To randomize for source colony, all newly emerged bees were collected into a single container prior to paint marking and subsequent placement into an observation colony. We marked and introduced approximately 500 bees per observation colony over a 9-day period (2–11 December 2015) using different paint colors to identify age. Observation hives were located at the Carl Hayden Bee Research Center, Tucson Arizona 32°16′30.1″ N 110°56′27.0″ W.

Kept in small greenhouses, the observation colonies (KA samples) were exposed to daily cycles of temperature and light, and provided foraging access to the local environment. As the observation colonies are a vertical linear arrangement of three frames, we provided a small radiant heat source when freezing temperatures were forecast, to compensate for the loss of thermoregulatory capacity possessed by a 3-dimemsional, full-sized colony. Every couple of days, we observed the behavior of known age bees for 10 minutes in the late afternoon to assess whether they had left the hive to collect resources. This behavior was verified by the presence of pollen on the legs of marked bees when returning to the interior of the hive, or waggle dancing on the comb to convey resource information to other hive members. We sampled 10 individuals per hive at 19, 33, 50, and 70 days of age. We roughly assessed total mortality in the KA samples as the estimated proportion of introduced bees surviving at 50 and 70-days. Samples from the KA observation hives were collected from 22 December 2015 (early winter) through 10 February 2016 (late winter), overlapping the time frame of warm winter sample collection from full-sized colonies (see below).

### 2.2. Warm vs. Cold Winter

In a separate related experiment, we hypothesized that different overwintering conditions would affect the hindgut microbiome. We sampled full-sized colonies kept in deep “Langstroth” hive boxes from both warm and cold overwintering environments. Warm winter (WW) colonies were sourced from a stationary apiary located East of Green Valley, AZ, USA in the Santa Rita Experimental Range 31°46′38″ N, 110°51′47″ W. These colonies were 6+ frames of bees at the beginning of the experiment. Worker bees of *Apis mellifera* subsp. *ligustica* were sampled at this location in early winter, mid-December 2015, and late winter, February 2016. From the edge of the brood nest, we sampled 16 colonies per time point for 32 total colony samples.

We sampled cold winter (CW) colonies from a migratory operation based in Jamestown ND, USA. From November through January, this operation places colonies in cold storage facilities at a constant 7 °C and 25% relative humidity with no access to environmental signals. Worker bees of *Apis mellifera* subsp. *carnica* were sampled just prior to entering the cold storage warehouse in Firth, Idaho, USA, 43°18′45″ N, 112°09′20″ W in early winter, mid-October 2015, and directly after they were removed from cold storage and transported to the almond orchards in late winter, mid-February 2016, near Snelling, California, USA, 37°31′33″ N, 120°31′52″ W. These colonies were 6+ frames of bees at the beginning of the experiment. From the edge of the brood nest, we sampled 16 colonies per time point for 32 total colony samples.

The nominal “sub-species” sampled from each location (CW and WW samples) interbreed freely, do not suffer outbreeding depression, and possess the same hindgut microbiome. Thus, we do not account for this genotypic variable in our design.

### 2.3. Tissue Samples and DNA Extraction

From the samples collected above, we dissected two neighboring hindgut tissues from the same individual worker bee for microbiota analysis of both the ileum and rectum. From the KA samples, we dissected 12 bees per collection time point for a total of 48 ileum and 48 rectum samples. From the CW and WW samples, we dissected one worker per time point, per sampled colony, resulting in 64 samples from each overwintering condition (WW or CW) comprised of 32 ileum samples and 32 rectum samples. Thus, as detailed below, we prepared 96 and 64/64 Illumina MiSeq libraries from the KA and WW/CW samples, respectively. This was also the initial sample size (*n* = 224) for both Bactquant and Fungiquant analysis (see below).

From both experiments, samples of individual bees were immediately frozen on dry ice and stored at −80 °C for next generation sequencing of the hindgut microbial communities. Immediately after being removed from −80 °C, individuals were surface sterilized and dissected in 95% ethanol using sterile forceps and dissection scissors. Dissected tissues were immediately placed into a 2 mL bead beating tube containing ~100 μL of 0.1 mm silica beads and 300 μL of 1X TE buffer (10 mM Tris-HCl, 1 mM EDTA) and immediately frozen on dry ice. All dissected tissues were stored at −80 °C. Prior to DNA extraction, each sample was bead beaten for a total of 2 min in 30 sec intervals. To each sample, 100 μL lysis buffer (20 mM Tris-HCl, 2 mM EDTA, 5% Triton X-100, 80 mg/mL lysozyme, pH 8.0) was added and the samples were incubated at 37 °C for 30 min. Total genomic DNA was further extracted using a Fermentas GeneJet Genomic DNA Purification Kit (#K0722) (Thermo Fisher Scientific, Waltham, MA, USA) following the protocol for Gram-positive bacteria.

### 2.4. PCR and MiSeq

The V6–V8 region of the 16S rRNA gene was amplified using universal (degenerate) PCR primers 799F (acCMGGATTAGATACCCKG) and bac1193R (CRTCCMCACCTTCCTC). Amplification was performed using the HotStarTaq Plus Master Mix Kit (Qiagen, Germantown, MD, USA) under the following conditions: 94 °C for 3 min, followed by 28 cycles of 94 °C for 30 s, 53 °C for 40 s and 72 °C for 1 min, with a final elongation step at 72 °C for 5 min. PCR products were confirmed using a 2% agarose gel. PCR products were then used to prepare DNA libraries following Illumina MiSeq DNA library preparation protocol. Sequencing was performed at the University of Arizona Genetics Core (UAGC) on a MiSeq following the manufacturer’s guidelines.

### 2.5. MiSeq Sequence Analysis

Sequences were processed using MOTHUR v1.43. [37]. Forward and reverse reads were joined using the make.contigs command. After the reads were joined, the first and last five bases pairs were removed using the SED command in unix. Sequences were then screened, using the screen.seqs command, to remove any sequences containing ambiguous bases. Unique sequences were generated using the unique.seqs command. A count file containing group information was generated using the count.seqs command. Sequences were aligned to Silva SSUREF database v102 [38] using the align.seqs command. Sequences not overlapping in the same region and columns not containing data were removed using the ilter.seqs command. Sequences were pre-clustered using the pre.culster command. Chimeras were removed using UCHIME [39] and any sequences that were not of known bacterial origin were removed using the remove.seqs command. All remaining sequences were classified using the classify.seqs command. All sequences with only one or two (single/doubletons) associated reads were removed using the AWK command in unix. A distance matrix was constructed for the aligned sequences using the dist.seqs command. Sequences were classified with the RDP Naive Bayesian Classifier [40] using a manually constructed training set containing sequences sourced from the greengenes 16S rRNA database [41], the RDP version 9 training set, and all full length honeybee-associated gut microbiota listed in NCBI. Operational taxonomic units (OTUs) were generated using the cluster command. Representative sequences for each OTU were generated using the get.oturep command (See Table S1).

### 2.6. Bacterial and Fungal Quantification

We used universal primer sets to amplify “total” fungal and bacterial communities from both the ileum and the rectum [42,43]. The bacterial 16S gene template was amplified using forward primer 27F (5′-AGAGTTTGATCCCTCAG-3′) and reverse primer 1522R (5′-AAGGAGGTGATCCAGCCGCA-3′). The fungal 18s gene template was amplified using forward primer PanFungal_18S_F (5′-GRAAACTCACCAGGTCCAG-3′) and reverse primer PanFungal_18S_R (5′-GSWCTATCCCCAKCACGA-3′). Plasmid vectors were created using Invitrogen’s pCRTM2.1 TOPOTM cloning vectors per the manufacture’s specifications. Ligated vectors were then transformed into DH5α™ cells per the manufacture’s specifications. Successfully, transformed colonies subsequently grown overnight in broth. Plasmid DNA was purified using a Thermo Scientific GeneJET Plasmid Miniprep Kit (#K0503). To determine 16S copy number for each sample we first calculated the mass of a single plasmid containing our insert using the Applied Biosystems equation. An Implen nanophotometer P300 was used to assess DNA concentration of the purified plasmid solution and subsequent 10-fold serial dilutions were made. We used these serial dilutions as standards for all subsequent qPCR quantifications. Total copy number was determined by first calculating the ‘raw’ copy number (nraw) in 1 µL of DNA based on the Cq value and the standard curve using the formula nraw = logarithmic trendline function. To determine the total number of copies present in each extraction, the nraw value was multiplied by the elution volume and any subsequent dilution(s). We compared the total bacterial and fungal abundance for both tissue types for the KA samples after log transforming for normality using a one-way ANOVA and Tukey–Kramer multiple comparisons correction. WW and CW samples were compared using *t*-tests.

### 2.7. Abundance Statistical Analysis

Microbial community structure and abundance were analyzed using three different approaches: MANOVA, Wilcoxon and repeated measures MANOVA. The MANOVA, performed on centered log ratios, accounts for microbiota structure while the Wilcoxon test examines absolute abundance changes in individual species or OTUs without regard to microbiota structure. The repeated measures MANOVA on centered log ratios examines the change in multiple dependent variables over time. We compared differences in the microbial community structure by known chronological age (KA samples) as well as within, and between the WW and CW environments.

### 2.8. Relative Abundance

The first and third approach analyzed the relative abundance of the raw amplicon data using a one-way MANOVA and repeated measures MANOVA. To allow the use of parametric multivariate analyses [44], we converted bacterial abundances to ratios among all OTUs [45] using the software CoDaPack’s centered log-ratio (CLR) transformation [46]. The CLR/MANOVA analysis computes a single vector that accounts for changes in the target taxa relative to the community as a whole [47]. To explore differences associated with overwintering condition, we examined the change in microbiota over time using repeated measures MANOVA, examining two time points, early and late winter. Pillai’s Trace test statistic was used for all MANOVAs to account for deviations in normality and homogeneity of covariance. Statistically significant MANOVA results were further analyzed with pairwise ANOVA tests followed by FDR correction for multiple comparisons.

### 2.9. Absolute Abundance

The second approach analyzed total cell number using pairwise Wilcoxon tests (Steel–Dwass correction for multiple comparisons). Wilcoxon tests do not account for the community as a whole and may be more applicable to taxa less influenced by community dynamics. The proportional abundance of OTUs returned by amplicon sequencing was multiplied by the total bacterial 16S rRNA gene copies determined with qPCR for each individual tissue type. To account for variability in 16S copy numbers across taxa the 16S total was divided by the number of 16s copy numbers associated with each core bacterial genome. All core bacteria contain four 16S rRNA gene copies except *L. kunkeei* (5), *B. asteroides* (2) and *Bombella apis* (1). Acetobacteraceae or ‘Alpha 2.1’ (copy number unknown) was designated a value of four, consistent with the copy number of closely related *Commensalibacter* genus (4). The copy number for Actinobacteria (2.9), Enterobacteriales (2) and Xanthomonadales (2.2) was determined using the average copy number for all genomes at the order level on NCBI. OTUs representing non-core diversity were summed (Σ OTUs 14-2667) and assigned a gene copy number of 4.2, the average for all bacteria [48].

## 3. Results

### 3.1. Microbial Community Analysis

**Figure 1:**
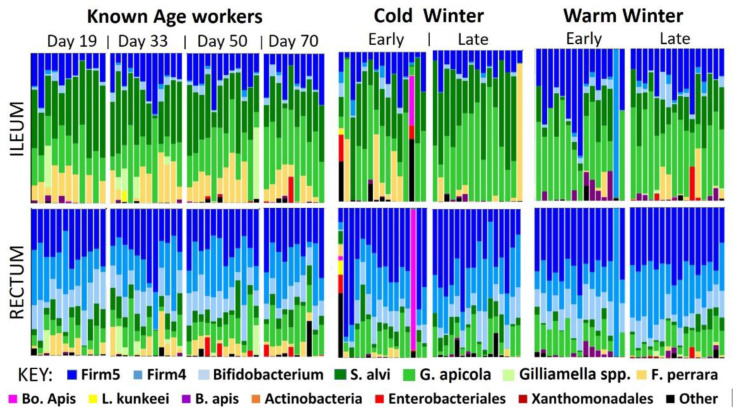
Bar charts representing the relative bacterial structure of each individual ileum and rectum. Known age (KA) samples (*n* = 47) were taken at 19, 33, 50, and 70 days of age from observation hives kept at Carl Hayden Bee Research Station AZ USA. The cold winter results are from 16 colonies sampled before and after 7 °C cold storage for 3 months in a large, climate controlled agricultural warehouse in Firth, ID, USA. Warm winter results are from 16 colonies kept outdoors at a warm southern location, with forage access near Green Valley, AZ, USA. Bacterial OTUs are identified by their respective color presented in the figure key. See supplementary material for more information.

Next-generation sequencing returned 7,084,800 quality trimmed reads (~400 bp in length) for the 220 amplicon libraries. Four of the 224 initial samples failed to result in usable data. Ileums were represented by 3,554,223 reads averaging 33,217 per library and rectums were represented by 3,530,577 reads averaging 32,391 per library. A total of 2667 OTUs were resolved at 98% similarity (Table S2). This threshold was used to distinguish between the similar 16S rRNA gene sequences of *G. apicola* and *F. perrara* at this gene loci. The top 13 OTUs and a 14th group consisting of ‘Other’ (Σ OTUs 14-2667), represented 97% and 3% of the total sequences, respectively, and were used for all statistical analyses. Mirroring previously described worker (nurse and forager) hindgut communities [28] the overwintering workers microbiota was taxonomically simple and represented by 10–13 OTUs (Figure 1).

### 3.2. Known Age Cohort

We hypothesized that the diutinus cohort in a southern environment would live long enough to transmit nutrition stored in their fat bodies to the first spring cohort. Counter to our hypothesis, not one of the KA marked bees we introduced to observation hives survived until spring. Only two of the four observation colonies survived through the winter. Although unquantified, at both the 17- and 33-day sampling time points (22 December and 5 January), there appeared to be enough marked bees per colony (estimated > hundreds) to satisfy the sampling effort through spring. However, at 50 days post-introduction (22 January), and considering only the two surviving observation colonies, the number of KA marked workers was estimated at 37 and 64, only 7 and 13% of the introduced total. Thus, we carefully tracked the observation hives following this sampling event to ensure a relevant sample size, and determine the timing of the final sampling event. At 70 days post introduction (11 February), there were just enough bees remaining alive to comprise a statistically relevant sample size, so we sampled all remaining bees from the two surviving colonies at 70 days of age.

Based on the KA samples, we determined age-related changes in the hindgut microbiota over a 70-day period of winter dearth. We report two approaches; (1) a MANOVA on CLR transformed data, considering OTU changes relative to the entire community, and (2) the Wilcoxon results, analyzing absolute abundance of OTUs without reference to other community members. The ileum microbiota remained unchanged over the 70-day period according to both metrics (Table S3) and relative abundance in the rectum was largely unchanged as well (ileum—Pillai’s trace = 0.90, F = 1.02, df = (39, 93), *p* = 0.46; rectum—Pillai’s trace = 1.09, F = 1.41, df = (39, 96), *p* = 0.09). In general, microbiota size changed significantly in the rectum, but microbiota structure (relative abundance) remained largely the same (Figure 1). In the rectum microbiota, five of six significant differences were based on absolute abundance. Five of the core bacteria—*Lactobacillus* Firm5 (*p* < 0.003), *Lactobacillus* Firm4 (*p* < 0.03), *B. asteroides* (*p* < 0.003), *S. alvi* (*p* < 0.003) and *G. apicola* (*p* < 0.02)—had greater absolute abundance in the 19- and 33-day samples compared to the 50-day samples (Table S4). Additionally, *Lactobacillus* Firm4 (*p* < 0.03) had greater absolute abundance in 19-day samples compared to 50-day samples. In contrast, the relative abundance of these microbiota members remained stable for both tissue types across all four age categories. The one exception to this pattern was an unknown *Gilliamella* spp. in the rectum that decreased with age from 33 to 70 days of age according to both absolute and relative abundance measures.

**Figure 2:**
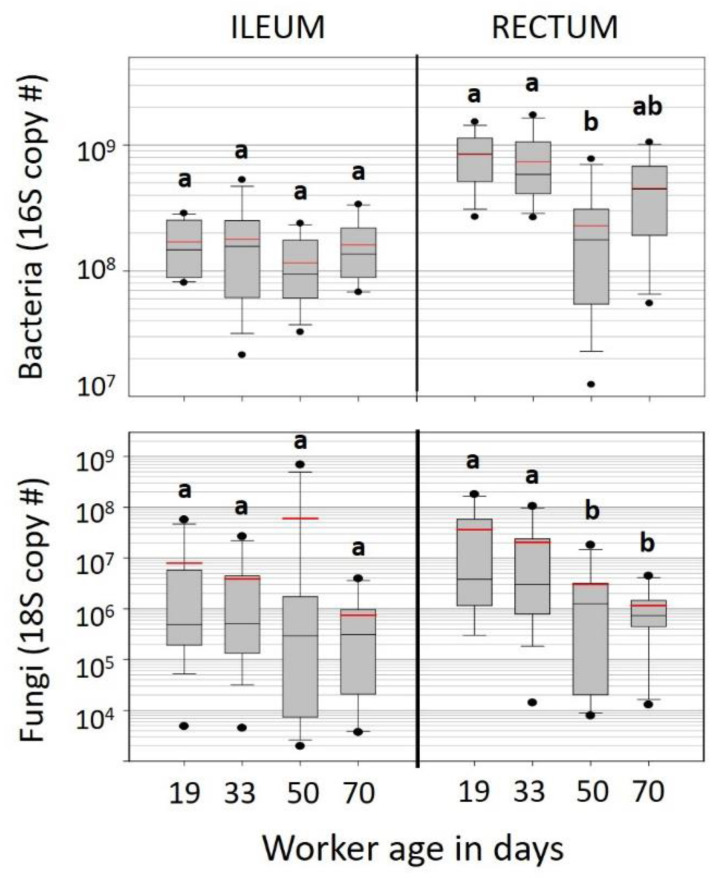
Box and whisker plots representing the total bacterial and fungal abundance associated with the known age (KA) ileum and rectal tissues. Workers sampled at 19, 33, 50, and 70 days of age. Box and whiskers represent 25–75% and 5–95% percent of the distribution, respectively. Each box and whisker plot represents 16 individual bee tissues. The y-axis is logarithmic scale (Log10). Different letters indicate significant differences (*p* < 0.05).

Both bacterial and fungal community size decreased significantly with known age in the hindgut. Based on Bactquant qPCR of the 16S rRNA gene [42], the size of the rectum microbiota decreased significantly with age, but the ileum microbiota remained the same size over time (Figure 2). Bacterial load in the rectum decreased continuously from 19 to 33 to 50 days, and then increased slightly at 70 days (Table S5). Based on Wilcoxon analyses, 50 day samples differed significantly from 19- and 33-day samples *p* < 0.009, (Figure 2). Similarly, fungal load decreased significantly with age in the KA rectums, with 19-day samples harboring greater fungal load (18s copy number) than both 50- and 70-day-old samples (*p* < 0.02 and *p* < 0.04, respectively; Figure 2, Table S5).

### 3.3. Repeated Measures Analysis CW and WW

We compared the change in the microbiota over time relative to overwintering environment. We found a significant multivariate interaction between time and overwintering environment for both the ileum (Pillai’s trace = 0.75, F = 3.8, df = (13, 16), *p* = 0.01), and the rectum microbiota (Pillai’s trace = 0.84, F = 6.4, df = (13, 16), *p* < 0.0001). Here, we report significant effects based on false discovery rate (FDR) for multiple hypothesis testing (see Table S6 for interaction plots and associated FDR). The majority of abundant OTUs remained remarkably stable from early to late winter regardless of overwintering environment. We report two strong interactions in low abundance OTUs. Relative to the cold indoor climate, an undescribed *Gilliamella* spp. increased significantly in the ileums (F1, 28 = 14.5, *p* = 0.0007) and rectums (F1, 28 = 30.6, *p* < 0.0001) of colonies from warm winter climates. Enterobacteriales remained unchanged in the CW environment, but increased significantly in the ileums (F1, 28 = 7.1, *p* = 0.01) and rectums (F1, 28 = 8.3, *p* < 0.007) of colonies from the WW climate.

### 3.4. Comparisons within and between CW and WW

Here we report significant differences within and between WW and CW environments in early and late winter according to relative and absolute abundance reporting MANOVA and Wilcoxon analyses, respectively (Tables S3 and S4). Within the indoor CW environment, the relative structure of the ileum and rectum microbiotas did not differ from early to late winter (Pillais trace *p* = 0.31 and *p* = 0.03, respectively). The microbiota wide test was marginally significant, such that no individual OTUs differed significantly following post hoc tests and application of the false discovery rate. Similarly, within the CW environment, the microbiotas of both tissues remained stable from early to late winter, with the exception of one low-abundance OTU; *Bombella apis* decreased in absolute abundance overwinter (*p* < 0.04; Table S3). Colonies from the WW environment differed significantly in relative abundance for both the ileum and rectum (Pillai’s Trace; *p* < 0.003 and *p* < 0.0001, respectively); low-abundance OTUs *Gilliamella* spp. and Enterobacteriales increased overwinter in both the rectum and ileum, while Xanthomonadales decreased in both the ileum and rectum.

Between the cold and warm winter environments, microbiotas of either tissue type did not differ in early winter, but many OTUs from both tissue types differed in late winter (Tables S3 and S4). According to both metrics, the unknown *Gilliamella* spp. and Enterobacteriales were more abundant in WW colonies in late-winter (*p* < 0.001 and *p* < 0.007, respectively). In both the ileum and rectum, Xanthomonadales (*p* < 0.0001) was at lesser relative abundance in WW environments with respect to other microbiota members, but did not differ considering absolute abundance. Highly evolved hive resident, *Bombella apis* was at greater absolute abundance in the ileums (*p* < 0.01) and rectums (*p* < 0.02) of WW colonies, Another hive resident, *L. kunkeei*, was at greater absolute abundance in the ileums (*p* < 0.04) of WW colonies. Typically, the most abundant bacterium in the hindgut, *Lactobacillus* firm5, was at lesser relative abundance in the rectums of WW colonies in late winter (*p* < 0.02, Table S4).

**Figure 3:**
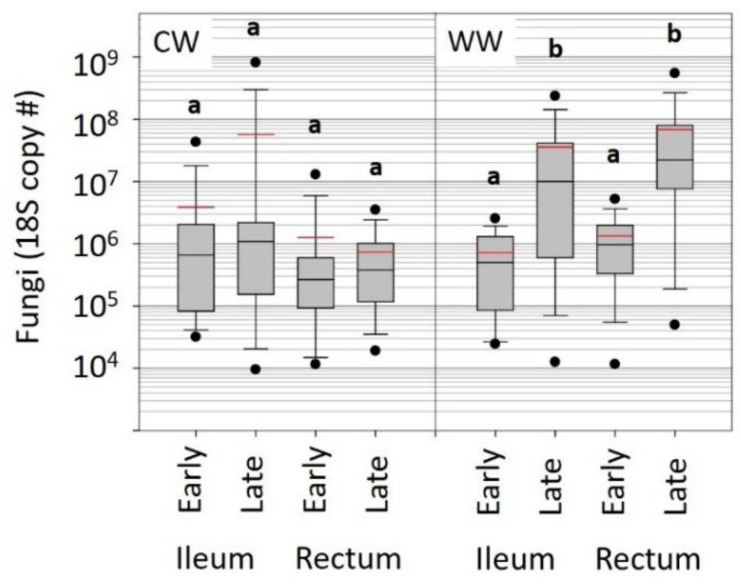
Box and whisker plots representing the total fungal abundance associated with cold winter (CW) and warm winter (WW) ileum and rectum tissues. Overwinter is depicted by early and late winter samples. Box and whiskers represent 25–75% and 5–95% percent of the distribution, respectively. The y-axis is a logarithmic scale (Log10). Different letters indicate significant differences.

In both tissue types, fungal (18S) abundance increased significantly from early to late winter in the WW colonies (*p* < 0.0001). Fungal abundance remained low and stable in the ileums and rectums of CW samples (Figure 3). There was no difference in total bacterial (16S) abundance between early or late WW/CW samples (Table S5).

**Figure 4:**
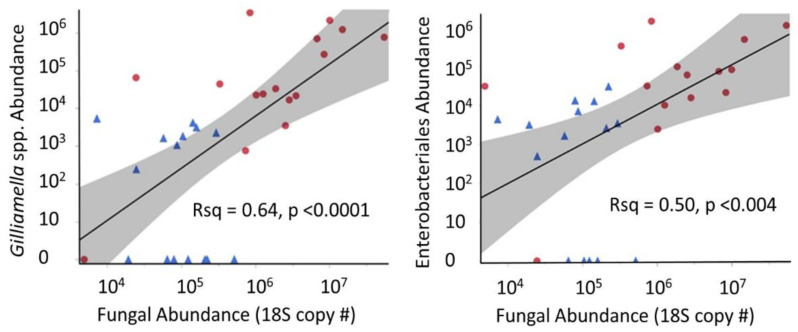
The significant association of bacterial and fungal abundance of two non-core bacterial OTUs overwinter in the rectum of worker bees from the warm winter (WW) environment. The *Gilliamella* species is unknown according to 16S sequence similarity, >3% dissimilar from both *G. apis* and *G. apicola*. The Enterobacteriales OTU is also uncharacterized. Blue triangles represent early WW rectum samples and red circles represent late WW rectum samples. Both axes are logarithmic scale (Log10).

Two of the bacterial groups that increased significantly in late winter WW samples were strongly and positively correlated with increasing fungal abundance across the assessed time period (Figure 4, *Gilliamella* spp.: Rsq = 0.64, *p* < 0.0001, and Enterobacteriales: Rsq = 0.50, *p* < 0.004).

## 4. Discussion

The beekeeping industry relies on properly overwintered colonies to meet early year pollination demands and replenish apiaries for the year to come [49]. Winter months, typically associated with extended periods of forage dearth, are endured by the diutinus (long-lived) worker phenotype [50,51]. This worker phenotype is a storage vessel of fats, proteins, and antimicrobial molecules, and lives many months in cold climates, but longevity in warm southern climates is unknown. Historically, beekeepers simply wintered their colonies locally outdoors, but climate controlled indoor wintering has been successful for large commercial beekeepers. Recent studies suggest that wintering colonies in cold climate controlled enclosures has numerous benefits [52,53]. However, considering the overwhelming evidence of its metabolic and physiological role, the extent to which winter climate effects the gut microbiome is relatively unknown. Our study had two interrelated goals; (1) to establish the longevity and age-associated hindgut microbial succession of diutinus workers in a southern climate, and (2) determine the effect of different overwintering environments on the diutinus hindgut microbiota.

We marked newly emerged worker bees, and introduced them to observation colonies to determine their longevity and microbiota characteristics overwinter. We found that worker aging was associated with smaller hindgut microbiotas that retained their relative structure (Figure 1). The ileum microbiota of aging diutinus workers remained stable in both size and structure from 19 to 70 days. The size of the rectum microbiota decreased with age but also retained its structure (Figure 2). Such structural stability across microbiota size variation suggests similar physiological function associated with the aging diutinus phenotype and may be related to the high Vg titers and antimicrobial nature of diutinus bees [8,13,32,54,55,56], though the degree to which physiological function relies on microbiota size or structure is still unknown. That the ileum microbiota remained stable, but the rectum microbiota decreased in size with age (Figure 2) may reflect the functional importance of the ileum microbiota, which has been linked to the maintenance of anoxic conditions in the hindgut and general host homeostasis [54]. When healthy (long-lived), the worker host may have more control maintaining the ileum microbiota relative to the rectum, and/or it is maintained in part by continuous consumption of simple sugars to fuel flight and thermoregulation.

Increasing chronological age was associated with significantly decreased bacterial and fungal load, but the relative structure of the ‘core’ hindgut bacteria did not change with age (Figure 1, Tables S3 and S4). Although the role of fungi in the rectum is unknown, it is generally accepted that an increased fungal load is unhealthy [53,57], and perhaps an indication that oxygen has become available in the typically anoxic rectum. Such increases are thought to result in dysbiosis, dysentery and subsequently reduced worker longevity and lower overall colony strength. The stasis of the hindgut microbiota seen in aging diutinus (KA) bees contrasts with OTU changes seen in more accelerated worker aging that occur during the behavioral transition from nurse worker to forager during the growth phase of the colony life cycle [26].

As the KA observation colonies and WW colonies were cared for similarly and overwintered in the same climate, combined information from these different experiments provides general insights into overwintering in warm southern climates. Most notably, diutinus worker longevity in southern climates is much less than that of diutinus workers in cold climates. That is, the oldest KA samples collected in the warm winter climate did not exceed 70 days of age, although a couple of the individuals sampled at 70 days may have survived slightly longer. Approximately 90% of introduced bees were dead (gone) by 50 days. Overwintering diutinus bees collected from cold winter climates typically exceed 135 days of age [8,10]. Colonies in southern climates are exposed to incoming pollen, cool nights (occasionally freezing) and warm days, and brood pheromone; confusing proximate signals that apparently override other signals to enter a state of energy conservation. Instead, these colonies continue low level foraging, egg laying and brood rearing behavior throughout the winter [7,32,58]. Indeed, throughout the entire overwintering experiment we observed many different marked bees aged 19–70 days of age returning with pollen or dancing to convey resource information in the observation colonies. Although warm winter foraging was greatly reduced relative to colony growth in the spring, the continuation of brood rearing behavior overwinter likely has an impact on colony-level energy conservation.

The observed early onset of mortality in the KA samples aids in understanding microbiome structure and fungal abundance of the WW samples and vice versa. The WW samples were a random assortment of worker bees, while KA samples were targeted sampling of known age workers. Although the KA cohorts were housed in observation hives that lack the three-dimensional structure required to facilitate thermoregulation, the aging (long-lived) bees appeared to harbor a healthier microbiome than did a randomly sampled subset of WW bees kept in full sized colonies under similar environmental conditions. Considering the rate of die-off and age-based demography observed in the KA samples, the random sampling of WW colonies would capture an aged-biased set of worker bees younger than 30 days old. Although colonies wintered in a warm climate are nutritionally depleted and experience an increase in pathogens [32], the ileum microbiota of KA workers did not change with age, remaining constant in both size and structure. Although workers living beyond 50 days represent a small proportion (<10%) of workers in the hive, these longer-lived bees were on a physiological trajectory that did not allow fungal proliferation (Figure 2). In contrast, we observed a stark increase in fungal abundance in both the ileum and rectum of the randomly sampled WW worker bees (Figure 3). Further, fungal abundance was correlated positively with an unknown *Gilliamella* spp. in the rectum (Figure 4). The opposite pattern was seen in KA bees. For that small subset of KA bees attaining “old age” (50–70 days), both fungal load and *Gilliamella* spp. abundance decreased significantly (Figure 2, Table S4).

*G. apicola* is metabolically associated with *S. alvi* in the ileum, but the broader *Gilliamella* spp. group contains highly variable function, and many species or strains are likely opportunistic [25,26]. Increased fungal load in WW bees was positively correlated with Enterobacteriales in the rectum (Figure 3). Here again, the role and/or impact of an increase in Enterobacteriales is unknown, however a few are known pathogens of honey bees (e.g., *Serratia* spp. [59]). Collectively, these data suggest that the warm winter climate influenced the colonies age demographics, and resulted in an increased fungal abundance in younger bees, ultimately influencing the microbiota structure. Whether the biological quantities reported here are sufficient to cause health deficiencies is unknown, but it is known that very few pathogen cells are often required for virulence. It has been demonstrated in honey bees that low-level infections with *Serratia marcescens* (Enterobacteriales) in the gut results in infection of the hemoceal and eventual septicemia [59].

In both warm and cold environments, the hindgut bacterial microbiota was generally stable over winter, with a few noteworthy exceptions (Figure 4). While microbiotas of colonies overwintered indoors at 7 °C remained stable, collective microbiota changes in the outdoor southern climate suggest compromised host physiology. Assuming that conservation of the ‘core’ microbial community during winter is an indicator of a true diutinus phenotype as we observed in the KA cohort, we detected multiple changes between the warm and cold winter microbiomes that suggest the CW indoor microbiota is healthier than the WW outdoor microbiota (Table S6). Although at low abundance, *Bombella apis, L. kunkeei*, Enterobacteriales and an unknown *Gilliamella* spp. were at lower absolute abundance in the late-winter CW samples compared to WW. These bacteria are not considered part of the core hindgut microbiota of workers, and may be early indicators of a compromised microbiota in the warm climate.

Fungal abundance in WW late-winter bees was almost two orders of magnitude greater than that of CW bees (Figure 3). This increased fungal load was positively correlated with non-core, likely opportunistic gut bacteria (Figure 4). Concerning changes in core hindgut bacteria, the CW indoor environment maintained a significantly greater ratio abundance of *Lactobacillus* firm5 in the rectum, perhaps providing greater resistance to opportunists and fungal invasion (Table S4). *Lactobacillus* firm5 is the most abundant hindgut bacterium, and likely has a major influence on the community as a whole, and host health. The structural stability of the microbiota observed in this and other studies suggest that shifts in the relative structure of the hindgut community may be biologically meaningful independent of statistical significance and absolute abundance. Providing both perspectives of microbial abundance offers greater context for the synthesis of novel information.

Our results suggest that the structure of the hindgut microbiota is intimately tied to host metabolism and colony-level energy conservation. In cold climates, colony brood rearing ceases and a single cohort of diutinus bees survives until spring, then transfers nutrition stored in their bodies to newly developing larvae. In general, the northern model of overwintering involves decreased immune expression in winter relative to summer, considered a strategy to conserve energy and increase overwintering survival [8]. Increased vitellogenin levels in long-lived diutinus bees may compensate to some degree for the decreased immune function documented in northern climates [8,13]. In contrast, we suggest that the southern model of overwintering is energetically costly. Energy is spent foraging, but the decreased diversity and quality of available winter forage likely contributes to pathogen susceptibility [60,61]. Nutrition necessary for the rearing of the year’s first cohort must be transferred through multiple (perhaps 2–3) ‘winter’ cohorts, with energy lost during each transfer. From a much larger colony-level study of southern overwintering [32], we detected a mean reduction in brood rearing, a net increase in nurse vitellogenin expression, but increased immune gene expression in winter relative to summer. Assuming that this study [32] approximates the gene expression in the present experiment, (see also [13]) the collective depiction of southern overwintering indicates minimal expression of the diutinus phenotype overwinter, more costly immune gene expression, and compromised energy conservation [32].

The current study and Kešnerová et al. (2019) [36] produced very different results, specifically with respect to the prevalence and abundance of *Bartonella apis*. Kešnerová et al. reports *Bartonella apis* as a predominant member of the overwinter hindgut microbiome, a novel finding in the honey bee system. Nearly all 16S amplicon based studies report sparse prevalence and low abundance of *Bartonella apis* regardless of experimental or environmental conditions [20,23,24,25,26,27,28,29,30,32,33,34]. Based on two separate climate conditions overwinter, our results do not support the trend for *Bartonella apis* to increase in overwintering bees, a pattern that dominated both overwintering sample sets from Kešnerová et al. [36]. However, we can only compare indirectly with this study because we lacked an outdoor cold winter control to determine the effect of daily temperature fluctuations associated with ambient but cold winter temperatures. Even so, our current study found *Bartonella apis* at 10% or greater abundance in only 2 of 110 samples. Further, both colony-level sampling experiments did not differ by *Bartonella apis* abundance regardless of overwintering condition. Although *Bartonella apis* did increase overwinter in CW relative to WW, the difference was not significant (Table S6). Finally, based on our KA cohort study conducted in ambient warm winter conditions, *Bartonella apis* was neither prevalent nor abundant and did not increase with age overwinter. In general, our study produced very different results than that of Kešnerová et al., but comparing the studies taxonomically and quantitatively is indirect given the separate environmental and molecular conditions.

Although the honey bee hindgut microbiota is highly predictable by OTU membership, a single study is unlikely to capture all the microbial variation associated with such a widespread host species. Many factors may contribute to differences among studies. Kešnerová et al. sequenced the microbiota with molecular primers specific to genera or species level. Their approach results in greater molecular specificity for binding sites, and less binding site competition relative to methods we used in this paper; universal bactquant primers [42] normalized by the relative abundance of *Bartonella apis* from an Illumina amplicon library produced using a different “universal” bacterial primer set. As suggested by the authors [36], there may be location-based differences including contributions from climate or bacterial strain variability. Kešnerová et al. (2019) [36] sampled winter bees from the tops of frames. Burritt et al. (2016) reported that >90% of sampled winter bees separated from their winter clusters, on the inner cover/tops of frames, were infected with *Serratia marcescens* in their hemolymph, a bacterium rarely detected in bees engaged in normal hive activities [62]. The geographic extent of this result is unknown, but suggests that the observed dominance of *Bartonella apis* may be influenced by targeted sampling within the hive. Additionally, for one of their major experiments, Kešnerová et al. (2019) [36] pooled 20 guts for each phenotype, whereas we examined individuals. Several recent studies indicate that when dysbiotic, the individual gut microbiome can increase substantially in size [34,35], such that a single dysbiotic individual could dominate the sampling results among 20 healthy individuals. Lastly, the microbiomes detailed by Kešnerová (2019) [36] may represent a recurrent enterotype because the non-winter seasons are dominated by *Gilliamella* with lesser amounts of *Lactobacillus* firm5 [27]. Most nurse/forager honeybee hindgut microbiomes sampled from non-winter are dominated by *Lactobacillus* firm5 [20,21,22,23,26,27,28,29,30,31,32,33,34,35]. Although both hindgut enterotypes are potentially healthy, they may react differently to perturbation or seasonal changes.

## 5. Conclusions

We determined the gut microbial succession of worker honey bees from a known age cohort. In a separate experiment, we sequenced the hindgut microbiota of workers subject to different overwintering environments. Our results suggest that the hindgut microbiota of diutinus (aging) workers remains stable with age, and resistant to fungal proliferation, consistent with recent studies that suggest an intimate link between honey bee bacterial microbiota structure and host central metabolism [29,63]. Different climates are known to alter colony behavior and physiology. We found that bees sourced from a warm climate as well as bees sourced from a cold indoor climate harbor a bacterial microbiota robust to the influence of environmental change. Based primarily on increased fungal abundance, we propose that warm outdoor wintering alters colony age demographics disrupting the stasis of the bacterial microbiota. Cold indoor conditions maintained a stable hindgut microbiota that resisted fungal proliferation and bacterial opportunism. While temperature may play an important role, many factors associated with colony organization, host physiology, and microbial taxa specific to location may contribute to the differences. The contrasting results of Kešnerová (2019) [36] suggest that the overwintering microbiota may vary extensively by region. Our results contribute to understanding overwintering honey bee biology and microbial ecology and provide insights into overwintering strategies. The extended stability of the gut microbiota demonstrated here provides additional support for the honey bee as a model system to study aging and host–microbe relationships.

## Data Availability

All sequence data were deposited in GenBank under Sequence Read Archive (SRA) accessions PRJNA705676 (WW and CW samples) and PRJNA705672 (KA samples).

## Supplementary Materials

The following are available online at https://www.mdpi.com/2075-4450/12/3/224/s1, Table S1: Bioinformatics commands, Table S2: Next-gen sequencing summary, Table S3: Ileum comparisons, Table S4: Rectum comparisons, Table S5: Bacterial–fungal abundance, Table S6: Repeated measures analysis.
